# Generation of Multipotent Foregut Stem Cells from Human Pluripotent Stem Cells

**DOI:** 10.1016/j.stemcr.2013.09.003

**Published:** 2013-10-10

**Authors:** Nicholas R.F. Hannan, Robert P. Fordham, Yasir A. Syed, Victoria Moignard, Andrew Berry, Ruben Bautista, Neil A. Hanley, Kim B. Jensen, Ludovic Vallier

**Affiliations:** 1 Wellcome Trust-Medical Research Council Stem Cell Institute, Anne McLaren Laboratory for Regenerative Medicine, Department of Surgery, West Forvie Site, Robinson Way, University of Cambridge, Cambridge CB2 0SZ, UK; 2 Wellcome Trust-Medical Research Council Stem Cell Institute, Tennis Court Road, Cambridge CB2 1QR, UK; 3 Wellcome Trust Sanger Institute, Hinxton CB10 1SA, UK; 4 Centre for Endocrinology and Diabetes, Institute of Human Development, Faculty of Medical and Human Sciences, Manchester Academic Health Sciences Centre, University of Manchester, Oxford Road, Manchester M13 9PT, UK

## Abstract

Human pluripotent stem cells (hPSCs) could provide an infinite source of clinically relevant cells with potential applications in regenerative medicine. However, hPSC lines vary in their capacity to generate specialized cells, and the development of universal protocols for the production of tissue-specific cells remains a major challenge. Here, we have addressed this limitation for the endodermal lineage by developing a defined culture system to expand and differentiate human foregut stem cells (hFSCs) derived from hPSCs. hFSCs can self-renew while maintaining their capacity to differentiate into pancreatic and hepatic cells. Furthermore, near-homogenous populations of hFSCs can be obtained from hPSC lines which are normally refractory to endodermal differentiation. Therefore, hFSCs provide a unique approach to bypass variability between pluripotent lines in order to obtain a sustainable source of multipotent endoderm stem cells for basic studies and to produce a diversity of endodermal derivatives with a clinical value.

## Highlights

• Multipotent foregut stem cells are derived from pluripotent cells
• Foregut stem cells can be expanded and retain their differentiation potential
• Foregut stem cells differentiate into liver and pancreatic cells
• Foregut stem cells decrease differentiation variability between cell lines

## Teaser

Hannan, Vallier, and colleagues have developed a defined culture system to differentiate and expand human foregut stem cells (hFSCs) from human pluripotent cells. hFSCs can self-renew while maintaining their capacity to differentiate into pancreatic and hepatic cells. hFSCs represent a sustainable source of multipotent endodermal cells for basic studies and can produce a diversity of endodermal derivatives with clinical value.

## Introduction

The development of a universal protocol to differentiate any hPSC line into a homogenous population of a specific cell type has been rendered difficult by the inherent variability that exists between lines. Epigenetic memory, inconsistent reprogramming, and genetic background are likely to be the main cause of this variability, which represents a major challenge for the development of personalized medicines (Lund et al., 2012) and for modeling diseases with a low-penetrance phenotype. The expansion of intermediate stages of differentiation could represent an attractive alternative to address this issue, especially if these cell types can be isolated from a heterogeneous population. For example, neuronal stem cells can be easily expanded from human induced pluripotent stem cell (hIPSC) lines differentiated toward the neuroectoderm lineage and then differentiated into a diversity of neurons, thereby bypassing the need to continuously grow pluripotent cells (Falk et al., 2012). However, the same approach with endoderm differentiation has been more problematic because the complex combination of inductive signals controlling the specification and patterning of this germ layer can be difficult to mimic in vitro (Sneddon et al., 2012). Following gastrulation, definitive endoderm migrates to form the primitive gut, which initially consists of a single flattened sheet of cells surrounded by mesoderm. Simultaneously, as migration is occurring, the primitive gut is becoming regionalized along the dorsal-ventral and anterior-posterior axes into foregut, midgut, and hindgut domains (Wells and Melton, 1999). This patterning is induced by the surrounding mesoderm, which secretes a variety of growth factors not limited to activin, Wnt, fibroblast growth factor (FGF), and bone morphogenetic protein (BMP) signal pathways (Deutsch et al., 2001; Zaret, 2002). The effects of these signaling pathways can be observed at the molecular level with distinct expression patterns of genes such as *SOX2* in the foregut and *CDX2* in the hindgut, while early *PDX1* expression marks the midgut (Zorn and Wells, 2009). It is from the foregut region that many of the major organ systems develop such as the liver, thyroid, lungs, the upper airways, the billary system, stomach, and pancreas. The induction of these organs is orchestrated by a complex combination of morphogen gradients secreted by other developing organ systems such as the cardiac mesoderm (Lemaigre and Zaret, 2004). The exact nature of these concentration gradients remains difficult to reproduce faithfully in vitro, but several groups (including our own) have made significant progress in understanding how different signaling pathways impact the foregut endoderm cells (Cho et al., 2012). To date, no one has described a foregut cell type that possesses the proliferative and differentiation potential observed within the early mammalian gut tube.

To address this challenge, we have developed a defined culture system to derive human foregut stem cells (hFSCs) from hPSCs. These stem cells can self-renew in vitro and resemble multipotent cells of the anterior primitive gut tube by their capacity to differentiate into pancreatic and hepatic cells. Furthermore, hFSCs can be easily derived from hIPSC lines resistant to endoderm differentiation, thereby enabling the production of endodermal derivatives from a broad number of hPSC lines.

## Results

### Anteroposterior Patterning of Definitive Endoderm Cells Generated from hPSCs Is Controlled by Activin/TGF-β and WNT Signaling Pathways

Our group has developed a defined culture system to direct the differentiation of hPSCs into a near-homogenous population of definitive endoderm (DE) cells that have the capacity to differentiate into hepatocytes and pancreatic progenitors (Brown et al., 2011; Cho et al., 2012; Rashid et al., 2010; Touboul et al., 2010; Vallier et al., 2009a; Yusa et al., 2011). Cells grown in these culture conditions successively express primitive streak markers (*T* and *Mixl1*), downregulate pluripotency markers (*NANOG*, *SOX2*, and *POU5F1*) and progressively upregulate definitive endoderm markers (*CXCR4*, *FOXA2*, *GATA4*, *CERB*, and *SOX17*) (Figures S1A–S1C available online). Flow cytometry analyses showed that 80% of the resulting DE population coexpresses CXCR4 and SOX17 (Figure S1D). Interestingly, the resulting population of DE cells is negative for genes marking the foregut (*SOX2*), the midgut/hindgut (*CDX2*), the pancreas (*PDX1*), the liver (*AFP*), and the lungs (*HOXA1*) (Figures S1E and S1F) This confirms that DE cells generated in vitro could correspond to early endoderm progenitor cells prior to anteroposterior patterning or organogenesis.

**Figure 1:**
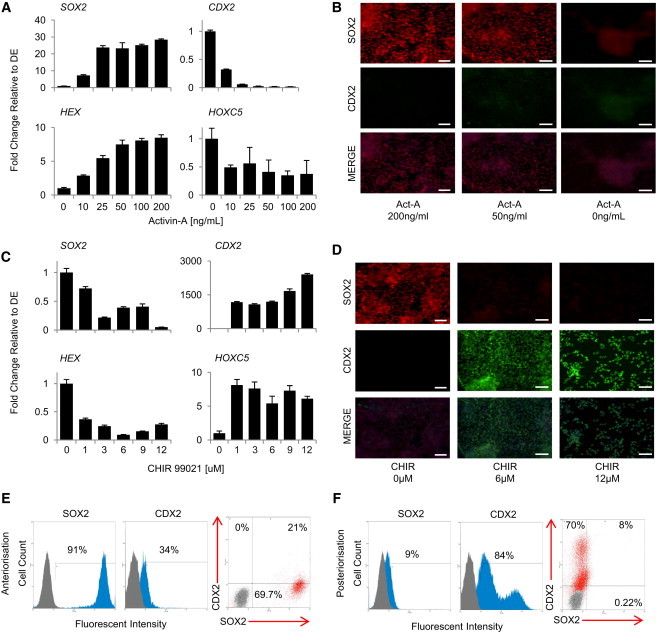
Activin and GSK3/WNT Control Anteroposterior Patterning of Definitive Endoderm In Vitro (A and B) Quantitative PCR (qPCR) and immunocytochemistry analyses show that DE cells grown for 5 days in the presence of activin-A express foregut anterior endoderm markers (*SOX2* and *HHEX*) without expressing hindgut markers (*CDX2* and *HOXC5*). (C and D) qPCR and immunocytochemistry analyses show that DE cells grown for 5 days in the presence of GSK3-beta inhibitor CHIR99021 express posterior endoderm markers (*CDX2* and *HOXC5*) without expressing anterior markers (*SOX2* and *HHEX*). (E and F) Fluorescence-activated cell sorting (FACS) analyses showing that DE cells derived from hESCs and grown for 5 days in the presence of activin express the anterior marker SOX2, while DE cells grown in the presence of CHIR99021 express the hindgut marker CDX2. Scale bars, 100 μm. Error bars represent SEM. See also Figure S1.

We next examined the capacity of DE cells to differentiate into representatives of the anterior and posterior domains of the primitive gut tube. We screened various growth factors (data not shown) and found that activin-A blocks *CDX2* expression while inducing expression of anterior gut markers such as *SOX2*, *HHEX*, and *HOXA3* (Figures 1A and 1B; data not shown). On the other hand, DE cells grown in the presence of the GSK3β inhibitor CHIR99021 express midgut/hindgut markers such as *CDX2* and *HOXC5* (Figures 1C and 1D) and show no expression of anterior markers. During both activin-A treatment and CHIR treatment, cells express high levels of the primitive gut markers GATA4, HNF4a, EpCAM, and HOXA2, demonstrating that cells retain their endodermal identity under these conditions (data not shown). Of note, flow cytometry analyses revealed that 90% of the cells express SOX2 after activin-A treatment while 85% of the cells were positive for CDX2 after CHIR99021 treatment (Figures 1E and 1F). Similar results were obtained using two hIPSC lines (BBHX8 and A1ATD.1) (Figures S2A–S2H). Taken together, these data show that activin-A and GSK3beta signaling direct the anteroposterior patterning of human DE in vitro.

### Hindgut/Midgut CDX2-Expressing Cells Generated from hPSCs Can Differentiate into Gut-like Organoids

**Figure 2:**
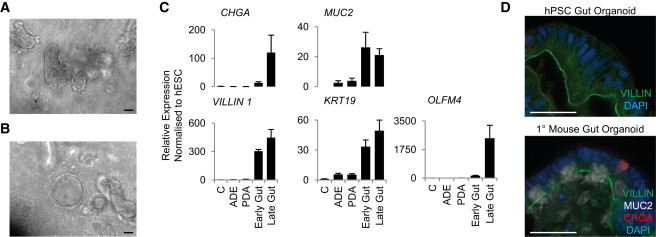
Hindgut Endoderm Generated from hPSCs Can Differentiate into Intestinal Derivatives When Grown in 3D Conditions (A and B) Hindgut CDX2-positive cells generated by growing DE cells in the presence of CHIR99021 for 5 days were grown in 3D cultures conditions to generate highly folded spheres resembling mature (A) and immature (B) gut organoids. (C) Accordingly, the resulting organoids could be grown for 2 months while progressively increasing the expression of gut markers (*OLFM4*, *CHGA*, *MUC2*, *Villin*, *and KRT19*). (D) Morphological similarities between gut organoid derived from hPSCs and primary mouse intestinal epithelial organoid cultures. Scale bars, 100 μm. Error bars represent SEM. See also Figure S1.

To further validate the identity of the cells generated in the presence of activin-A or CHIR99021, we decided to test their capacity to differentiate into intestinal cells. SOX2^+^ cells and CDX2^+^ cells were grown into 3D organoid culture conditions (Spence et al., 2011) known to promote posterior gut differentiation. SOX2^+^ cells grown under these conditions ceased to proliferate and could not be expanded (data not shown), whereas CDX2^+^ cells formed spheroids with highly folded structures resembling intestinal epithelium (Figures 2A and 2B). Gut organoids expressed intestinal markers (mucin, villin, and chromagranin A) and could be expanded for more than 2 months while displaying a progressive increase in the markers of adult intestinal epithelium (Figure 2C). Finally, comparative immunocytochemistry analysis of CDX2^+^ cell-derived organoids with primary mouse intestinal organoids demonstrated a polarized epithelium with apical villin expression in both types of organoids (Figure 2D). These data confirm that CDX2^+^ cells generated in the presence of CHIR99021 have the differentiation potential to form midgut/hindgut progenitors, while activin-A-induced SOX2^+^ cells have lost this capacity. These SOX2^+^ cells could consequently be equivalent to foregut progenitors (Arnold et al., 2011).

### Foregut Cells Generated from hPSCs Can Be Expanded for a Prolonged Period of Time in Defined Culture Medium

**Figure 3:**
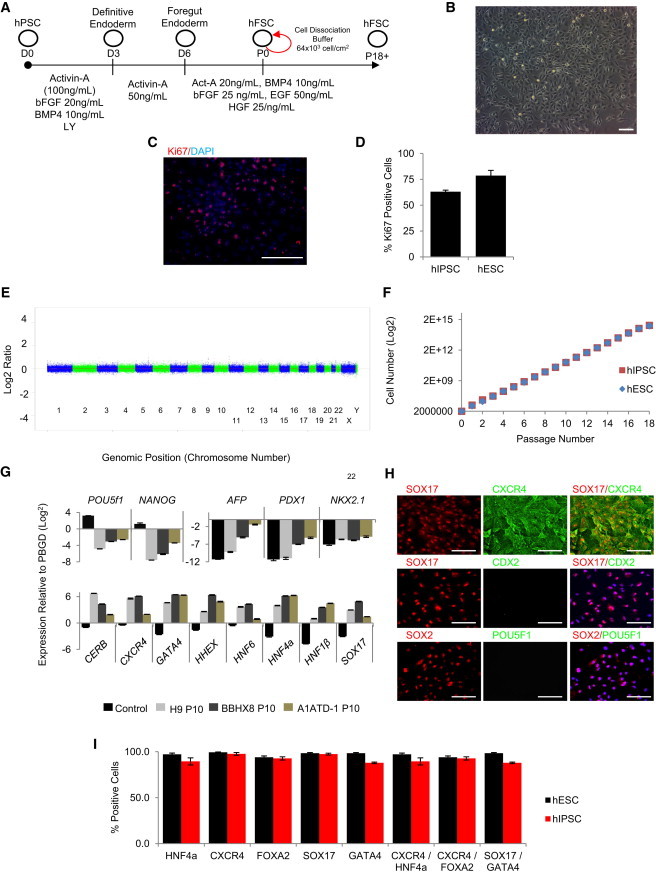
hPSC-Derived Foregut Can Self-Renew In Vitro (A) Method to differentiate and to propagate Foregut Stem Cells derived from hPSCs. (B) Bright-field image of foregut stem cells growing as an epithelium. (C and D) After ten passages, approximately 80% of hFSCs express Ki67. (E) CGH profile of late-passage hFSCs showing no change in copy number. (F) Growth curve of hFSCs. (G and H) qPCR and immunocytochemistry analyses showing that hFSCs derived from hESCs (H9) and hIPSCs (BBHX8 and A1ATD-1) can be grown for more than ten passages while maintaining the expression of foregut markers (*CERB*, *HNF1β*, *HNF6*, *CXCR4*, *GATA4*, *HHEX*, *HNF4α*, and *SOX17*). The expression of pluripotency (*POU5F1, NANOG*), pancreatic (*PDX1*), hepatic (*AFP*), lung (*NKX2.1*), and gut (*CDX2*) was not observed during propagation. (I) A near-homogenous population of hFSCs derived from both hESC- and hIPSC-coexpressing foregut genes was evident after ten passages. (C), (D), and (I) represent the number of positive cells counted from ten random immunocytochemistry fields in three experiments with a minimum of 100 cells per field normalized to the DAPI count. Scale bars, 100 μm. Error bars represent SEM. See also Figure S3.

We then tested the capacity of foregut SOX2^+^ cells to self-renew in vitro. DE cells derived from human embryonic stem cells (hESCs) were differentiated for 4 days in the presence of activin-A and were subsequently cultured in the presence of a diverse combination of growth factors (data not shown). Following this approach, we identified that the combination of activin-A, basic fibroblast growth factor (bFGF), BMP4, hepatocyte growth factor (HGF), and epidermal growth factor (EGF) was sufficient to expand foregut SOX2^+^ cells for more than 20 passages (Figure 3A). DE cells grown under these conditions form a simple epithelium (Figure 3B) that can be serially passaged at a plating density of 40–50 × 10^6^ cells/cm^2^ of plate surface area. Foregut cells grown in these culture conditions express homogenously the proliferation marker Ki67 (Figures 3C and 3D), retained a normal karyotype (Figure 3E), and after 20 passages have an expansion potential of more than 4.2 × 10^8^-fold (Figure 3F). Importantly, these culture conditions were only supportive of foregut cells, because significant cell death was observed with neuronal-like and fibroblast-like cells. This selection could explain how a near-homogenous population of foregut cells could be easily obtained only after few passages (Figure S3A). After more than ten passages in these culture conditions, foregut SOX2^+^ cells did not express transcripts or proteins of pluripotency (*POU5f1* and *NANOG*), lung (*NKX2.1*), hepatic (*AFP*), or pancreatic (*PDX1*) markers while maintaining the expression of foregut markers (*HNF4α*, *SOX17*, *CXCR4*, *EpCAM*, *HNF1β*, *GATA4*, *Cer*, *SOX2*, *HNF6*, and *HNF1beta*; Figures 3G, 3H, and S3B–S3D). Flow cytometry analyses showed that SOX17 and CXCR4 were coexpressed near homogenously (Figure S3A) along with other foregut markers such as CXCR4/HNF4a (>90% double positive), CXCR4/FOXA2 (>90% double positive), and SOX17/GATA4 (>90% double positive; Figure 3I). Importantly, similar results were obtained with two hIPSC lines (BBHX8 and A1ATD.1; Figures S3A–S3D) (Brown et al., 2011; Rashid et al., 2010; Vallier et al., 2009a). Finally, we observed that foregut cells could be frozen after five passages and then thawed to be expanded for at least five additional passages without any loss in gene expression profiles or observable loss of proliferation capacity (data not shown and Figure S4A). Together, these data demonstrate that our culture system captures a homogenous population of foregut cells that can self-renew in vitro and are lineage restricted to endodermal tissue and thus could be representative of an endodermal stem cell (referred thereafter as human foregut stem cells or hFSCs).

### hFSCs Can Differentiate into Cells Expressing Hepatic, Pancreatic, and Lung/Thyroid Markers

**Figure 4:**
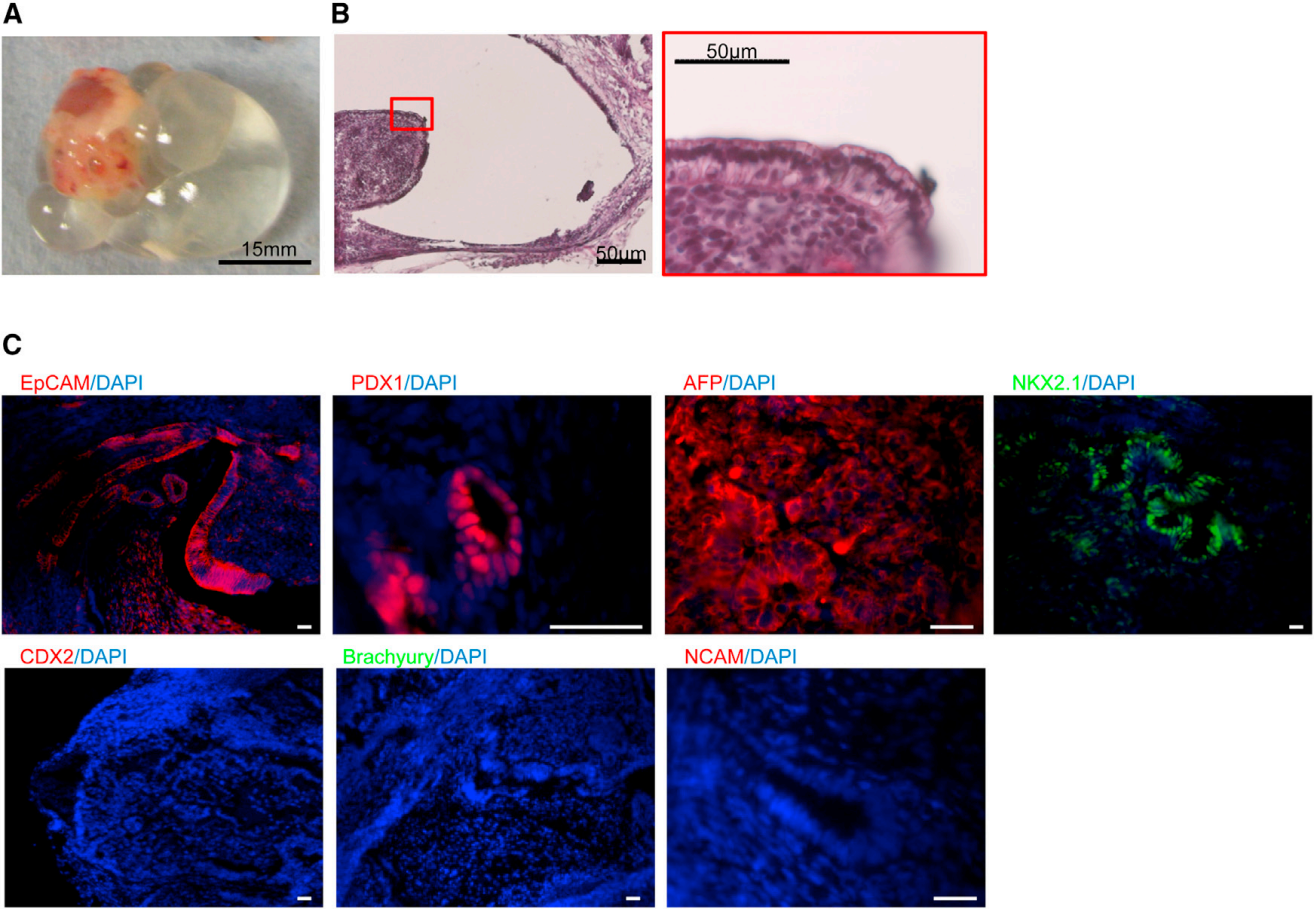
hFSCs Do Not Form Teratomas and Differentiate into Cells of the Foregut Only (A) Large cystic hFSC outgrowth under the kidney capsule of a NOD-SCID mouse. (B) Cryosection of a hFSC outgrowth showing large cystic structures lined with epithelial cells. (C) Immunocytochemistry showing foregut outgrowths expressing EpCAM, PDX1, AFP, and NKX2.1. Scale bars, 100 μm or 50 μm as shown. See also Figure S4.

To further characterize hFSCs, we decided to investigate their spontaneous capacity to differentiate in endodermal derivatives. For that, 1.0 × 10^5^ hFSCs were transplanted under the kidney capsule of nonobese diabetic severe combined immunodeficiency (NOD-SCID) mice and incubated in vivo for 10 weeks. Both hESC- and hIPSC-derived hFSCs produced very large cysts with very limited solid outgrowth (Figure 4A). Histological examination revealed that hFSCs did not produce teratomas, as there were no tissues representative of the ectoderm and mesoderm lineages. Instead, the outgrowths were formed mainly of cyst resembling glandular tissue and lung epithelium (Figure 4B). Accordingly, immunostaining analyses indicated that a large majority of cells contained in hFSCs-derived outgrowth expressed the lung/thyroid marker Nkx2.1, while fewer pockets of cells expressing liver (AFP/EpCAM) and pancreatic marker (PDX1) could also be detected (Figure 4C). However, cells expressing mesoderm (Brachyury) or neuroectoderm (N-CAM) markers were never observed and the hindgut marker CDX2 was also absent (Figure 4C) Together, these data suggest that hFSCs have the capacity to differentiate into liver, pancreas, and lung/thyroid cells and thus could be considered as multipotent stem cells.

**Figure 5:**
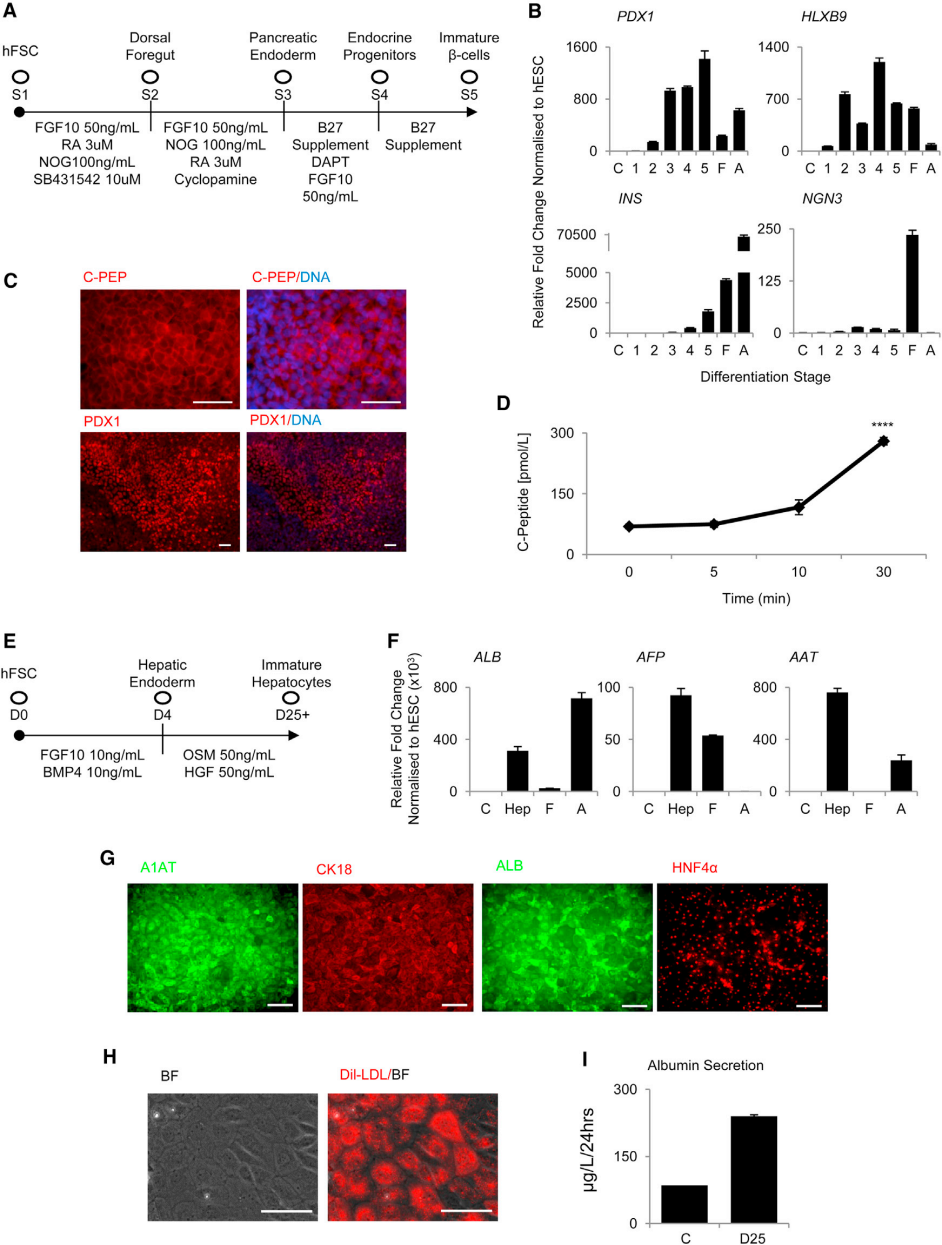
hFSCs Have the Capacity to Differentiate into Multiple Derivatives of the Foregut (A) Method to differentiate hFSCs into pancreatic cells. (B) hFSCs grown in these culture conditions progressively express pancreatic bud markers (*PDX1* and *HLXB9*) and then endocrine markers (*INS* and *NGN3*). F, fetal pancreas; A, adult pancreas. (C) C-peptide and PDX1 expression was confirmed by immunocytochemistry of cells differentiated for 25 days. (D) Day 25 pancreatic cells released insulin into the medium when treated with high-glucose Dulbecco’s modified Eagle’s medium over 30 min. (E) Method used to differentiate hFSCs into hepatic cells. (F) After 25 days of differentiation, cells expressed hepatic markers (*ALB*, *AFP*, and *A1AT*). C, undifferentiated hESC; Hep, hFSC-derived hepatocytes; F, fetal liver; A, adult liver). (G) Expression of mature hepatocyte markers (ALB, A1AT, CK18, and HNF4α) was confirmed by immunocytochemistry. (H and I) Hepatocytes were capable of uptaking LDL from the medium (H) and secreted albumin (I). C, medium with no cells. Scale bars, 100 μm. ^∗∗∗∗^p < 0.0001. Error bars represent SEM. See also Figures S4–S6.

To confirm this hypothesis, we tested the ability of hFSCs to differentiate into liver and pancreatic cells using culture systems recently developed by our group to produce these cells types directly from hPSCs (Cho et al., 2012; Rashid et al., 2010). hFSCs grown in culture conditions inductive for pancreatic specification (Cho et al., 2012) (Figure 5A) sequentially expressed early pancreatic markers (*HLXB9* and *PDX1*), then endocrine progenitor marker (*NGN3*), and finally beta cell marker (c-peptide/insulin) (Figure 5B). As reported previously, more than 90% of cells were PDX1 positive at stage 4 of the pancreatic differentiation protocol (Figures S4B and S4C) (Cho et al., 2012). After 25 days of differentiation, cells expressing c-peptide, PDX1, glucagon (GCG), and somatostatin (SST) could be observed by immunocytochemistry and c-peptide release was detected upon glucose stimulation (Figures 5C and 5D). Importantly, approximately 15%–20% of pancreatic cells were positive for C-peptide and 10%–20% of these C-peptide-positive cells were polyhormonal (i.e., coexpressing both C-peptide and either GCG [20%] or SST [10%]) (Figure S4D–S4G). While surprising, these result are in agreement with our previous study showing that monohormonal/glucose-responsive cells could be generated from hPSCs using a defined culture system (Cho et al., 2012). Importantly, further characterization beyond the scope of the current study will be necessary to validate the real functionality of the insulin-expressing cells generated under our culture conditions. Similar analyses were performed using our liver differentiation protocol and hFSCs described previously (Hannan et al., 2013; Rashid et al., 2010; Yusa et al., 2011) (Figure 5E). Under these conditions, hFSCs rapidly differentiate into a near-homogenous population of hepatocyte-like cells expressing liver markers (*AFP*, *ALB*, *A1AT*, HNF4α, and CK18; Figure 5F and 5G). In addition, a vast majority of hepatocyte-like cells (90%) coexpressed albumin and ASGPR and alpha-1-antitrypsin as reported previously (Figures S4H–S4K) (Rashid et al., 2010; Hannan et al., 2013). Further functional characterization also showed that these cells could uptake low-density lipoprotein (Figure 5H) and cardiogreen from tissue culture medium (Figure S6) and secrete AAT and albumin (data not shown and Figure 5I). Importantly, multiple hFSC lines derived from different hIPSC lines displayed similar differentiation efficiency (Figures S5 and S6), while frozen/thawed hFSCs could also generate liver and pancreatic cells. Indeed, defrosted hFSCs could be expanded for five passages and then differentiate efficiently into cells expressing pancreatic (PDX1 > 90%) and hepatic (AFP > 90%) markers (Figures S4J–S4M). Taken together, these data demonstrate that hFSCs have the capacity to differentiate into foregut derivatives including lung/thyroid, pancreatic, and hepatic cells, thereby confirming that they are multipotent.

### Clonal Differentiation of hFSCs Confirms Their Multipotency

**Figure 6:**
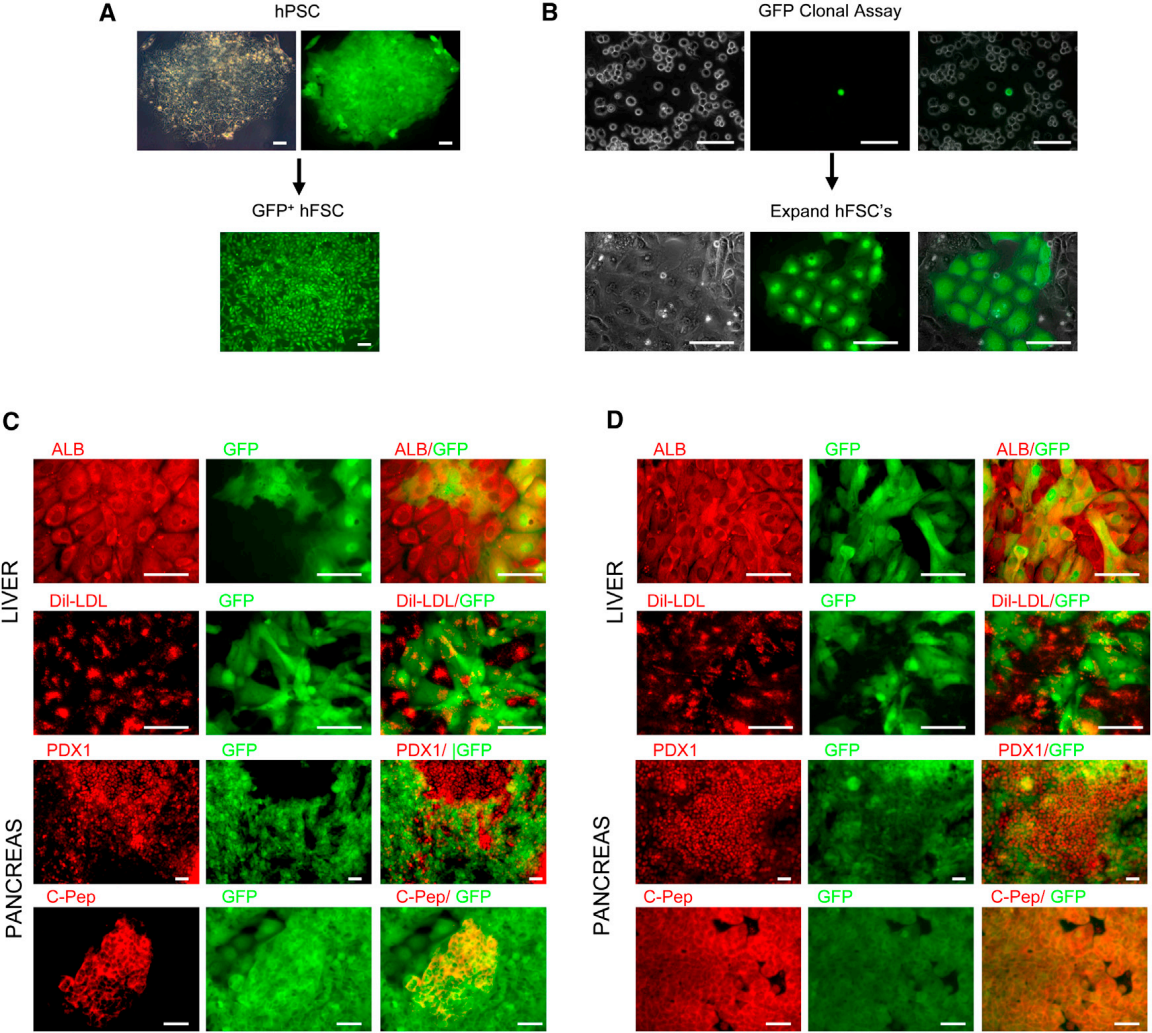
Single hFSCs Are Multipotent (A) GFP-expressing hPSCs were differentiated into hFSCs. (B) Single GFP-positive hFSCs were seeded onto a layer of non-GFP hFSCs and then expanded for five passages. The resulting population was then split into culture conditions inductive for liver or pancreatic differentiation. (C and D) GFP-hFSCs differentiated for 25 days were found to respectively generate cells expressing liver markers (ALB, LDL-uptake) and pancreatic markers (PDX1, C-peptide) from both hESC-derived (C) and hIPSC-derived (D) hFSCs. Scale bars, 100 μm.

To further reinforce these results, we decided to confirm that single hFSC are multipotent. hFSCs grow as an epithelium and single-cell isolation systematically resulted in cell death, thereby excluding conventional clonal assays. To bypass this limitation, we generated GFP-expressing hFSCs that were dissociated into single cells and individually placed on a well of a 24-well plate containing non-GFP-expressing hFSCs (Figures 6A and 6B). The following day, wells containing a single GFP-positive hFSC were marked for expansion, and after five passages, the resulting cells were differentiated into hepatic and pancreatic cells. Of note, such an approach, while not strictly clonal, did allow us to follow the expansion of a single GFP cell and assess the differentiation potential of its progeny. Indeed, GFP-positive hFSC cells grown in these respective culture conditions expressed hepatic (ALB, A1AT, AFP, and HNF4a and low-density lipoprotein [LDL] uptake) and pancreatic (PDX1, INS, NGN3, and SST) markers, thereby providing the necessary evidence that hFSCs generated from hESCs and hIPSCs (data not shown; Figures 6C and 6D) are multipotent stem cells.

### Generation of hFSCs Bypasses Variability between hIPSC Lines

**Figure 7:**
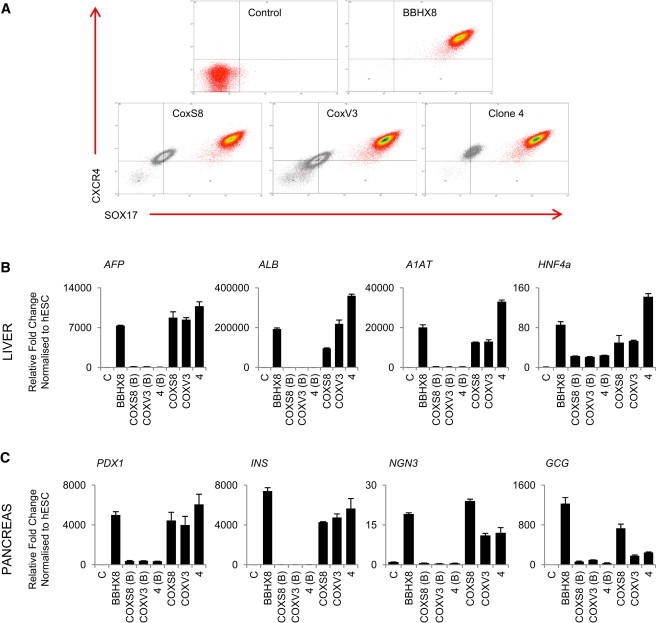
Generation of hFSCs Overcomes Variability between hIPSC Lines (A) FACS analyses showing the fraction of cells coexpressing the endoderm/foregut markers SOX17/CXCR4 before isolation (colored plot) and after expansion (passage 5, black plot) of hFSCs generated from hIPSC lines with high (BBHX8) and low (CoxS8, CoxV3, and Line4) endoderm differentiation capacity. Secondary only antibody only control, gray population. (B and C) hIPSCs with low endoderm capacity of differentiation cannot differentiate into liver or pancreatic cells [COXS8(B), COXV3(B), 4(B)], while hFSCs generated from the same cells seven passages later can differentiate into cells expressing markers for hepatocytes (*A1AT*, *AFP*, and *ALB HNF4a*) and pancreatic cells (*GCG*, *PDX1*, *INS*, *NGN3*) at levels comparable to positive control (BBHX8). Error bars represent SEM.

hIPSC lines can be easily generated from a broad number of patients, and this has opened the possibility to model a diversity of disorders for basic studies and drug screening. However, recent reports have shown that hIPSC lines can vary in their capacity to generate cell types with clinical value. The precise mechanisms responsible for this variability remain unclear but are likely to combine several origins, including epigenetic memory, genetic background, and abnormal reprogramming. This variability also presents a major challenge for personalized cell-based therapy and large-scale genetic studies, because each hIPSC line might require protocol optimization. Accordingly, we recently analyzed the capacity of 48 hIPSC lines derived from 16 individuals to differentiate into endoderm, and while a majority of hIPSC lines could differentiate in our culture system, we identified three endoderm-resistant hIPSC lines (COXS8, COXV3, and Line4). These lines produced less than 30% of SOX17-positive DE cells (Figure 7A). Importantly, this heterogeneity precluded further differentiation toward the pancreatic/hepatic lineages. We therefore decided to isolate hFSCs from these endoderm-resistant hIPSC lines. For that, heterogeneous endoderm cells generated from the hIPSC lines COXS8, COXV3, and Line4 were grown for 3 additional days in the presence of activin-A to promote foregut specification and the resulting cells were transferred into culture conditions supporting hFSCs expansion. Interestingly, contaminating cells of nonendodermal origin stopped proliferating and progressively disappeared upon passaging. Flow cytometry analyses show that cells grown for five passages homogenously expressed SOX17 and CXCR4 (99%) similarly to hFSCs generated from hIPSCs proficient for endoderm production (BBHX8) (Figure 5A). Therefore, our culture system selectively amplifies hFSCs even when they originate from a heterogeneous population of DE cells. The resulting population of hFSCs was expanded for two additional passages and then transferred into culture conditions inductive for pancreas and liver differentiation. Cells differentiated toward the liver lineage expressed hepatic markers (*AAT*, *ALB*, *AFP*, and *HNF4α*) at a level similar to hepatocyte-like cells generated from control hFSCs (Figure 5B). Similarly, cells differentiated toward the pancreatic lineage cells expressed *PDX1*, *INS*, and *NGN3* (Figure 5C). Together, these results show that hFSCs can be easily generated from hIPSCs with reduced endoderm differentiation capacity, enabling the production of hepatic and pancreatic cells. Therefore, derivation of hFSCs could be achieved from numerous hIPSC lines and enable us to bypass in part the variability of hIPSC lines to generate hepatic/pancreatic cells.

## Discussion

Derivation of foregut stem cells with a strong proliferative capacity as well as the ability to self-renew represents an attractive cell source for multiple applications within the regenerative medicine field, including disease modeling, developmental studies, and cell-based therapies. Accordingly, our results describe a stepwise method to differentiate hPSCs into a multipotent population of foregut stem cells. Importantly, production of foregut cells has been reported previously (Green et al., 2011), but our study provides a culture system allowing the isolation, expansion, and differentiation of multipotent self-renewing foregut stem cells. Similarly, a recent study has shown that multipotent DE cells could be expanded in vitro (Cheng et al., 2012; Sneddon et al., 2012), yet these cells express a broad diversity of markers that render their embryonic identity difficult to establish. Furthermore, these studies relied on feeders, Matrigel, 3D culture conditions, or serum, any of which is not compatible with large-scale or clinical applications. Nevertheless, hFSCs resemble mouse anterior definitive endoderm cells isolated from mouse embryonic stem cells using the combination of reporter gene for *HEX* expression and the cell surface marker CXCR4 (Morrison et al., 2008). In agreement, hFSCs display a similar gene expression profile including the expression of HHEX and CXCR4 and share a similar capacity of differentiation toward liver and pancreas. However, hFSCs could also have the unique capacity to generate lung/thyroid cells while they have lost their capacity to generate gut cells. More importantly, hFSCs can be easily isolated from a diversity of hPSCs lines without the need for cell sorting and complex genetic modifications, thereby allowing the production of a near-homogenous population of cells with clinical value. Overall, the hFSC culture system addresses several important limitations associated with current methods available to isolate and to expand endodermal stem cells.

hFSCs also share fundamental characteristics with their in vivo counterpart, including the expression of key markers such as FOXA2, CXCR4, HHEX, SOX17, and CERB. Nevertheless, the exact type of foregut cell described here is yet to be fully defined, as lineage tracing experiments have shown that foregut may contain only bipotential progenitors able to differentiate toward the hepatic and pancreatic lineages (Deutsch et al., 2001). However, the property of in vivo progenitors is likely to be dictated by their localization within the foregut and thus their surrounding environment. Moreover, the gut tube initially possesses a high degree of plasticity. Indeed, the hindgut domain, if taken at an early time point, is capable of producing liver and pancreatic bud structures when either juxtaposed against foregut cardiac mesoderm or placed in culture conditions with BMP and FGF (Bossard and Zaret, 2000; Wells and Melton, 2000). This suggests that during the early stages of gut formation, the entire gut epithelial sheet could be multipotent. Thus, the culture system described here could be less restrictive, enabling hFSCs to display the full range of their developmental plasticity.

Self-renewing and multipotent adult stem cells could represent an advantageous source for the generation of large quantity of “safer” differentiated cells required for cellular therapy, because they could strongly reduce the risk of teratomas associated with pluripotent stem cells. However, it is important to underline that the isolation and serial passage of hFSCs did not improve the overall maturity of the end-stage population after differentiation, whether this be liver, lung, or pancreatic cells. Indeed, hepatocytes or pancreatic cells generated from either freshly derived foregut cells or P10 hFSCs still display a combination of adult and fetal characteristics and are not fully functional with regard to cytochrome P450 activity or insulin secretion, respectively. These results are in agreement with a broad number of studies that have demonstrated that fetal-like pancreatic/hepatic cells can be efficiently generated from hPSCs (Cho et al., 2012; Hannan et al., 2013; Rashid et al., 2010; Touboul et al., 2010) or from organ-specific progenitors (Cardinale et al., 2011; D’Amour et al., 2006; Deutsch et al., 2001; Morrison et al., 2008; Si-Tayeb et al., 2010; Sneddon et al., 2012; Sullivan et al., 2010; Van Haute et al., 2009; Wang et al., 2013; Zhao et al., 2009). Thus, the current report does not claim to solve this major challenge or even to improve existing protocol of differentiation. Our study only establishes that hFSCs are able to produce liver and pancreas cells similar to those generated from endodermal cells directly produced from hESCs (Cho et al., 2012; D’Amour et al., 2006; Hannan et al., 2013; Rashid et al., 2010; Si-Tayeb et al., 2010; Sneddon et al., 2012; Sullivan et al., 2010; Touboul et al., 2010; Van Haute et al., 2009; Zhao et al., 2009). The generation of fully functional cells from hPSCs remains a distant goal that will require the development of innovative approaches far beyond the scope of this work (Shan et al., 2013; Takebe et al., 2013).

To conclude, expansion of a multipotent foregut progenitor population is of considerable interest with regard to clinical applications. Indeed, our culture system is compatible with large-scale production of a near-homogenous population of endodermal cells that could greatly simplify the production of cells for cell-based therapy. Furthermore, derivation of hFSCs allowed for differentiation of all the tested hIPSC lines without the need to establish individual protocols. Therefore, hFSCs not only provide a unique in vitro model of human development but also represent an important tool to deliver the clinical promises of hIPSCs in the field of personalized medicine.

## Experimental Procedures

### Generation of hIPSCs

hIPSCs (BBHX8 and A1TATD) were derived using retrovirus-mediated reprogramming of human skin fibroblasts using the Yamanaka factors as described elsewhere (Rashid et al., 2010). Approval for the collection of human tissue samples was obtained from the North West Ethics Committee (13/NW/0205). All animal work was carried out with full home-office approval (project license 80/2397).

### Generation of GFP hPSCs and Clonal Analyses

GFP-expressing H9, BBHX8, and A1ATD-1 cells were generated by stable transfection using lipofectamine 2000 (Invitrogen) as described previously (Vallier et al., 2001). GFP-positive cells were differentiated into foregut cells and then dissociated into single cells. An individually isolated GFP cell was then transferred into a well containing non-GFP-positive hFSCs. Wells were visually inspected 12 hr after plating, and wells containing a single GFP-positive hFSC were selected for clonal expansion.

### Human Embryonic Stem and Induced Pluripotent Stem Cell Culture

hESCs (H9) and hIPSCs (BBHX8, A1ATD-1, COXV3, COXS8, Line4, and IPS40) were cultured in a chemically defined, feeder-free culture system as described previously using activin-A (10 ng/ml) and bFGF (12 ng/ml) (Brown et al., 2011; Rashid et al., 2010; Teo et al., 2011; Vallier et al., 2009a; Vallier et al., 2009b). Cells were passaged every 7 days using a mixture of collagenase IV or collagenase and dispase at a ratio of 1:1.

### Differentiation of hPSCs into Endoderm

Cells were differentiated into definitive endoderm using CDM-PVA and activin-A (100 ng/ml), BMP4 (10 ng/ml), bFGF (20 ng/ml), and LY294002 (10 μM) for 3 days as described previously (Cho et al., 2012; Rashid et al., 2010; Vallier et al., 2009a; Yusa et al., 2011).

### Patterning of Definitive Endoderm

DE cells were cultured in RPMI+B27 medium with activin-A (50 ng/ml) for 3-4 days to generate foregut cells. DE cells were cultured in RPMI+B27 medium with CHIR99021 (6 μM) for 4 days to generate posterior endoderm.

### Differentiation of Posterior Endoderm into 3D Gut Organoids

Posteriorized endodermal cells were embedded in growth-factor-reduced Matrigel (BD Biosciences) containing B27 supplement (RA depleted) (Invitrogen), human R-spondin (500 ng/ml) (R&D), human Noggin (100 ng/ml) (R&D), human EGF (100 ng/ml) (R&D), and Jagged-1 peptide (1μM) (AnaSpec). Cell/Matrigel mix was overlayed with Advanced Dulbecco’s modified Eagle’s medium (DMEM)/F12 (Gibco) supplemented with 2 mM GlutaMax (Invitrogen), 10 mM HEPES (Invitrogen), and 100 U/ml penicillin per 100 μg/ml streptomycin containing B27 supplement (RA depleted) (Invitrogen), Y-27632 (10 μM) (Sigma Aldrich), Noggin (100 ng/ml) (R&D), human EGF (100 ng/ml) (R&D), human R-spondin (1 μg/ml) (R&D), and human Wnt3a (100 ng/ml) (R&D).

### Passaging and Maintenance of hFSCs

hFSCs were cultured on gelatine coated plates prepared as described for hPSC maintenance in RPMI medium containing B27 Supplement, NEAA, penicillin/streptomycin, activin-A (10 ng/ml), bFGF (20 ng/ml), BMP (10 ng/ml), HGF (20 ng/ml), and EGF (50 ng/ml). Cells were passaged every 4–7 days using cell dissociation buffer (CDB). Cells were washed once with PBS then incubated in CDB at 37°C for 10–15 min. Cells were scraped as small clumps and transferred to a 15 ml tube and centrifuged at 800 rpm for 2 min. Cells were washed once with RPMI medium and then resuspended in RPMI medium containing the cocktails of growth factors described above and ROCK inhibitor Y-27632 (10 μM). Cells were plated at a density of 40–60 × 10^3^ cells/cm^2^ of plate surface area. ROCK inhibitor was not used during subsequent days of culture. Medium was changed the following day and every subsequent day until cells were 80%–90% confluent.

### Differentiation of hFSCs into Hepatic Endoderm

Hepatic differentiation has been described previously (Cho et al., 2012; Rashid et al., 2010; Yusa et al., 2011). Briefly, hFSCs were cultured in RPMI + B27 containing BMP4 (10 ng/ml) and FGF10 (10 ng/ml) for 4 days. Cells were then cultured in hepatocyte basal medium (Lonza) containing oncostatin M (50 ng/ml) and HGF (50 ng/ml) for at least an additional 20 days.

### Differentiation of hFSCs into Pancreatic Endoderm

hFSCs were differentiated into pancreatic endoderm using a five-step process as described previously (Cho et al., 2012). During stage 1 (S1), hFSCs were cultured in Advanced DMEM (Invitrogen) supplemented with SB-431542 (10 μM; Tocris), FGF10 (50 ng/ml; AutogenBioclear), all-trans retinoic acid (RA, 2 μM; Sigma) and Noggin (50 ng/ml; R&D Systems) for 3 days. For stage 2 (S2), the cells were cultured in Advanced DMEM supplemented with human FGF10 (50 ng/ml; AutogenBioclear), all-*trans* retinoic acid (RA, 2 uM; Sigma), KAAD-cyclopamine (0.25 uM; Toronto Research Chemicals), and Noggin (50 ng/ml; R&D Systems) for 3 days. For stage 3 (S3), the cells were cultured in human FGF10 (50 ng/ml; R&D Systems) for 3 days. For maturation of pancreatic progenitors (stage 4 [S4]), cells were grown in Advanced DMEM + 1% vol/vol B27 and DAPT (1 mM) for 3 days and for 3 additional days in Advanced DMEM + 1% vol/vol B27 (stage 5 [S5]). During S4 and the final stage (S5) of differentiation, medium devoid of insulin was used so as not to interfere with immunocytochemistry and ELISA assays. Additionally, only antibodies raised against C-peptide were used to avoid potential false-positive results.

### Immunocytochemistry

For a complete list of primary and secondary antibodies, please refer to Table S1. hPSCs or their differentiated progenitors were fixed for 20 min at 4°C in 4% paraformaldehyde and then washed three times in PBS. Cells were incubated for 20 min at room temperature in PBST (0.1% Triton X-100; Sigma; in PBS) containing 10% donkey serum (Serotec) and subsequently incubated overnight at 4°C with primary antibody diluted in 1% donkey serum in PBST. Cells were then washed three times in PBS and incubated with secondary antibodies in 1% donkey serum in PBST for 2 hr at room temperature. Unbound secondary antibody was removed by three 5 min washes in PBS. Hoechst 33258 was added to the first wash.

For immunocytochemistry on 3D organoids, organoids were removed from Matrigel, fixed in 4% paraformaldehyde, and embedded in 4% agarose before processing for paraffin sections. Following antigen retrieval, samples were permeabilized with 0.5% Triton X-100 and blocked in 10% fetal bovine serum before overnight incubation in primary antibody. Samples were washed with PBS and incubated with secondary antibodies for 1 hr at room temperature before being counterstained using DAPI. Samples were imaged using a Zeiss Imager M.2, equipped with AxioCam MRm and MRc cameras and AxioVision software for image capture.

Immunocytochemistry on cryosections was performed by drying sections at 37°C for 30 min before antigen retrieval at 95°C. Cells were then fixed in 4% paraformaldehyde for 20 min before they were blocked and permeabilized using a solution of 10% donkey serum 0.01% Triton X-100 for 30 min. Primary and secondary antibodies were applied using a 1% donkey serum 0.001% Triton X-100 solution.

### Flow Cytometry

For a complete list of primary and secondary antibodies used for flow cytometry, please refer to Table S1. Adherent cells were washed twice in PBS and then incubated for 20 min at 37°C in cell dissociation buffer (Invitrogen). Cells were dissociated by gentle pipetting and resuspended at approximately 0.1–1 × 10^6^ cells/ml in PBS. Cells were pelleted and fixed by resuspending cells in 4% paraformaldehyde solution at 4°C for 20 min. Cells were washed in PBS and then blocked and permeabilized in PBS containing 10% donkey serum and 0.01% Triton X-100. Cells were then incubated in a solution of 1% donkey serum 0.001% Triton X-100 containing the primary antibody. Cell were incubated for at least 2 hr at room temperature or overnight at 4°C. Cells were then washed three times in PBS 1% donkey serum and incubated with secondary antibodies in for 2 hr at room temperature. Unbound secondary antibody was removed by three to five washes in PBS. Cells were then analyzed using a FACS Calibur machine (BD Biosciences). All flow cytometry experiments were gated first using unstained cells and then cells containing the secondary antibody only. On all flow cytometry plots, the secondary-only population is shown in gray. All gates shown on scatterplots and histogram plots were set to the secondary-only control. All flow cytometry was validated with immunocytochemistry to ensure that false-positive or false-negative results were not recorded.

### ELISA

hESCs grown for 25 days in culture conditions inductive for pancreatic specification were cultured in differentiation medium without insulin for 24 hr prior to glucose stimulation. Cells were washed three times with PBS and preincubated in DMEM supplemented with 2.2 mM glucose referred to as “low glucose” conditions (Invitrogen) for 60 min at 37°C. Preincubated cells were grown in DMEM containing 22 mM glucose, referred to as “high glucose conditions” for 5, 10, or 30 min. Supernatants were collected for determination of C-peptide release. ELISA analyses were performed using the Mercodia Ultrasensitive C-peptide ELISA kit (Mercodia).

For albumin secretion assays, high binding surface COSTAR 96-well plates (Corning) were coated overnight with affinity-purified rabbit polyclonal antibodies against albumin (Abcam 87564) at 2 μg/ml in carbonate/bicarbonate buffer (Na2CO3/NAHCO3, pH 9.5). After washing (0.9% w/v NaCl, 0.05% v/v Tween 20), the plates were blocked for 2 hr in blocking buffer (PBS, 0.25% w/v BSA, 0.05% v/v Tween 20). Culture medium was diluted in blocking buffer, and 50 μl was added to each well and then incubated for 2 hr. After washing, the wells were incubated with corresponding monoclonal antibodies (1 μg/ml diluted in blocking buffer) and incubated for 2 hr. Bound monoclonal antibodies were detected with rabbit anti-mouse immunoglobulin G horseradish peroxidase-labeled antibody (Sigma Aldrich, 1:20,000) for 1 hr. The reaction was developed with TMB liquid substrate (Sigma Aldrich) for 10 min in the dark and the reaction was stopped with 1 M H_2_SO4. Absorbance was read at 450 nm on a Thermo-max microplate reader (Molecular Devices).

### Uptake of LDL

The Dil-LDL staining kit was purchased from Cayman Chemicals and the assay was performed according to the manufacturer’s instructions.

### Statistics

All experiments were carried out at a minimum of three technical triplicates. Data represent biological triplicates. All values are expressed as the mean ± SEM. Differences between means were assessed by t test using Graph Pad Prism 6 software. p < 0.05 was considered significant. All p values are indicated in figure legends.

## Author Contributions

N.R.F.H. conceived of and designed the study, performed experiments, developed protocols and validation, collected and interpreted data, produced figures, and wrote and edited the manuscript. R.P.F. performed 3D gut organoid differentiation experiments, collected and interpreted data, and edited the manuscript. Y.A.S. performed cryosectioning and staining experiments and interpreted data. V.M. collected data and edited the manuscript. R.B. performed CGH analysis and interpreted data. N.A.H. and A.B. obtained human fetal primary tissue samples. K.J. interpreted gut organoid data and edited the manuscript. L.V. conceived of and designed the study, interpreted data, and edited the manuscript. All authors read and approved the final version of the manuscript.

## Supplemental Information

Document S1. Supplemental Experimental Procedures and Figures S1–S6

Table S1. List of Primary and Secondary Antibodies Used, Related to Figures 1, 2, 3, 4, 5, 6, and 7

Table S2. List of qPCR Primer Sequences Used, Related to Figures 1, 3, 5, and 7
