# Supplementary material for: Generation of Multipotent Foregut Stem Cells from Human Pluripotent Stem Cells
Source: Stem Cell Reports. 2013 Oct 10;1(4):293–306. doi: 10.1016/j.stemcr.2013.09.003 (PMC3849417; doi:10.1016/j.stemcr.2013.09.003)
Supplement: Document S1. Supplemental Experimental Procedures and Figures S1–S6 [file mmc1.pdf]

Stem Cell Reports, Volume *1*

Supplemental Information

Generation of Multipotent Foregut Stem Cells

from Human Pluripotent Stem Cells

Nicholas R.F Hannan, Robert P. Fordham, Yasir A. Syed, Victoria Moignard, Andrew Berry, Ruben Bautista, Neil A. Hanley, Kim B. Jensen, and Ludovic Vallier

Inventory of Supplementary Data

S1. Complements Figure 1 and shows the initial differentiation step to produce DE cells.

S2. Complements Figure 1 and shows that the data in Figure 1, obtained using hESC can be reproduced using two different hIPSC lines.

S3. Complements Figure 3 and shows that the basic profile of hFSC's can be reproduced using two different hIPSC lines.

S4. Complements Figure 4 & 5 by showing extra data of the characterisation of hFSC's specifically their ability to undergo cryopreservation, retain their foregut gene expression profile and then produce progenitor cells. This figure also addresses the issue of polyhormonal cells.

S5. Complements Figure 5 demonstrating that the pancreatic differentiation can be reproduced in two different hIPSC lines.

S6. Complements Figure 5 showing that the hepatic differentiation can be reproduced using two different hIPSC lines

Supplementary Table 1. Antibody list.

Supplementary Table 2. PCR Primer sequences.

Supplementary Methods. Provides additional methodologies used in this study

Supplementary Figure Legends:

Supplementary Figure 1. Differentiation of hPSCs into a near homogenous population of Definitive Endoderm cells. (**a**) hESCs grown in the presence of high dose of Activin, BMP4, FGF2 and PI3Kinase inhibitor down regulate pluripotency markers *(POU5f1, SOX2, NANOG*), transiently express primitive streak markers (*T, MIXL1*) and express endoderm markers (*HHEX, CERB, CXCR4, SOX17*). (**b**) Pluripotent cells express pluripotency markers (POU5f1, NONOG, SOX2) and do not express DE markers (SOX17,FOXA2). (**c**) DE cells generated after 3 days of differentiation express CXCR4, SOX17, GATA4 and FOXA2 and do not express markers of pluripotency POU5f1 and NANOG. (**d**) hESCs derived DE cells express SOX17 and CXCR4 by flow cytometry. Grey population = secondary only control. (**e**) DE cells do not express specific markers of the foregut (*SOX2*) or hindgut/midgut endoderm (*CDX2*). (**f**) Similarly DE cells do not express detectable levels of markers for pancreas (*PDX1*), liver (*AFP*) or lung (*HOXA1*). Scale bars = 100µm

Supplementary Figure 2. DE derived from hIPSC can also be patterned into foregut and hindgut endoderm in vitro. (**a-d**) Activin and GSK3-beta inhibition (CHIR99021) drive differentiation of DE generated from hIPSC line BBHX8 into foregut and hindgut respectively. (**a**) QPCR showing Activin causes upregulation of *SOX2* and *HHEX* while down regulating *CDX2* and *HOXC5* while DE cells grown in the presence of CHIR do not express *SOX2* while up regulating the hindgut markers *CDX2* and *HOXC5*. The ubiquitous gut marker *HOXA2* is not differentially expressed between the two conditions. (**b**) Immunocytochemistry analyses showing that DE cells generated from hIPSC line BBHX8 cultured in the presence of CHIR express EpCAM, CDX2 and HNF4a and are negative for Sox2. (**c**) FACS analyses showing that BBHX8 differentiate homogenously toward a foregut population ($SOX2^+/CDX2^-$) or (**d**) hindgut populations ($CDX2^+/SOX2^-$) when grown in the presence of Activin or CHIR respectively. (**e-h**) Effect of Activin-A and GSK3 beta inhibition on the hIPSC line A1ATD-1. (**e-f**) QPCR and immunocytochemistry showing that activin induces expression of foregut genes (*SOX2, HHEX*) while repressing hindgut genes (*CDX2, HOXC5*) and GSK3 beta inhibition represses foregut gene expression while promoting hindgut gene expression. (**g-h**) FACS analysis showing the efficiency of A1ATD-1 DE in forming a foregut population ($SOX2^+/CDX2^-$) (**g**) and a hingut population ($SOX2^-/CDX2^+$) (**h**). Scale bars = 100µm

Supplementary Figure 3. hFSCs can be grown as an homogenous population of cells in vitro. (**a**) FACS analyses showing that SOX17 and CXCR4 are homogenously expressed in hFSCs derived from hESCs (H9 P3, H9 P10) or hIPSCs (BBHX8 P10, A1ATD.1 P10). (**b**) hFSC derived from hESCs and cultured for more than 10 passages are negative for genes marking differentiation toward pancreas (PDX1), Lung (NKX2.1, TBX1), and Liver (AFP) while maintaining the expression of foregut markers such as EpCAM, CXCR4 and HNF4a. Similarly hFSCs derived from hIPSC lines BBHX8 (**c**) and A1ATD.1 (**d**) grown for 10 passages express foregut markers (SOX2, SOX17, EpCAM, CXCR4, HNF4a) and do not express markers specific for pluripotency (POU5f1), pancreas (PDX1), liver (AFP), lung (NKX2.1, TBX1) and hindgut (CDX2). Scale bars = 100µm

Supplementary Figure 4. hFSC's can be cryostored and retain their capacity to differentiate into mature hepatic and pancreatic cells. (**a**) hFSC's passaged to P5 and cryopreserved for 2 weeks, thawed and passaged to P10 express foregut genes CXCR4, SOX17, FOXA2, GATA4 and HNF4a. Population analysis of D25 pancreatic cells derived from hESC (**b**) or hIPSC (**c**) hFSC's showing the percentage of c-peptide and PDX1 positive cells. (**d,e,**) Population analysis of polyhormonal cells in D25 pancreatic cultures from hESC derived hFSC showing the percentage of c-peptide and glucagon (**d**) and c-peptide and somatostatin (**e**) double positive cells. Population analysis of polyhormonal cells in D25 pancreatic cultures derived from hIPSC derived hFSC's showing somatistatin/c-peptide double positive cells (**f**) and glucagon/c-peptide double positive cells (**g**). Population analysis of D25 hepatocyte cultures from hESC derived hFSC's (**h**) and hIPSC derived hFSC's (**i**) showing percentage of ASGPR, AAT and ALB positive cells. (**j-m**) Population analysis of cryopreserved hFSC's derived from hESC (**j**) or hIPSC (**k**) at P10 showing percentage of cells expressing lung (NKX2.1), liver (AFP) and pancreas (NKX2.1) markers. Immunocytochemistry for progenitor populations from hESC (**l**) and hIPSC (**m**) derived hFSC's. All data points b-c and h-k represent the number of positive cells counted from 10 random ICC fields of at least 100 cells normalised to the DAPI count. Data points d-g were normalised to the total number of hormonal cells present and represent a percentage of total hormonal cells present in 10 random ICC fields containing at least 100 cells from 3 experimental replicates. Scale bars = 100μm

Supplementary Figure 5. hFSCs can differentiate into pancreatic endoderm. (**a**) hFSC derived from hIPSC line BBHX8 differentiate into pancreatic cells expressing *HLXB9, PDX1, NGN3 and INS*. (**b**) Immunocytochemistry showing expression of PDX1, C-Peptide and NGN3. (**c**) Insulin ELISA showing a detectable release of insulin into tissue culture medium after 10 and 30 minutes stimulation with high glucose DMEM. (**d**) hFSC's derived from the hIPSC line A1ATD-1 differentiate into pancreatic cells. (e) Immunocytochemistry confirms expression of C-peptide, PDX1 and NGN3. (**f**) Insulin ELISA shows that stimulation of D25 pancreatic cells with high glucose medium results in detectable release of insulin after 10 and 30 minutes. Scale bars = 100µm * = $p<0.05$ ** = $p<0.01$ **** $p<0.0001$

Supplementary Figure 6. hFSC derived from hPSCs differentiate into hepatic endoderm. (**a**) hFSCs derived from hIPSC line BBHX8 were differentiated for 25 days in culture conditions inductive for hepatic differentiation and the resulting cells express hepatic genes (*A1AT, AFP, ALB, HNF4a*) and (**b**) showed functional activity such as LDL and cardiogreen uptake from tissue culture medium. (**c**) Similarly hepatic cells cultured for 25 days derived from the hIPSC line A1ATD-1 express hepatic genes and (**d**) show functional characteristics such as LDL and cardiogreen uptake. (F = foetal liver, A = adult liver). Scale bars = 100µm

Hannan et al Supplementary Figure 1

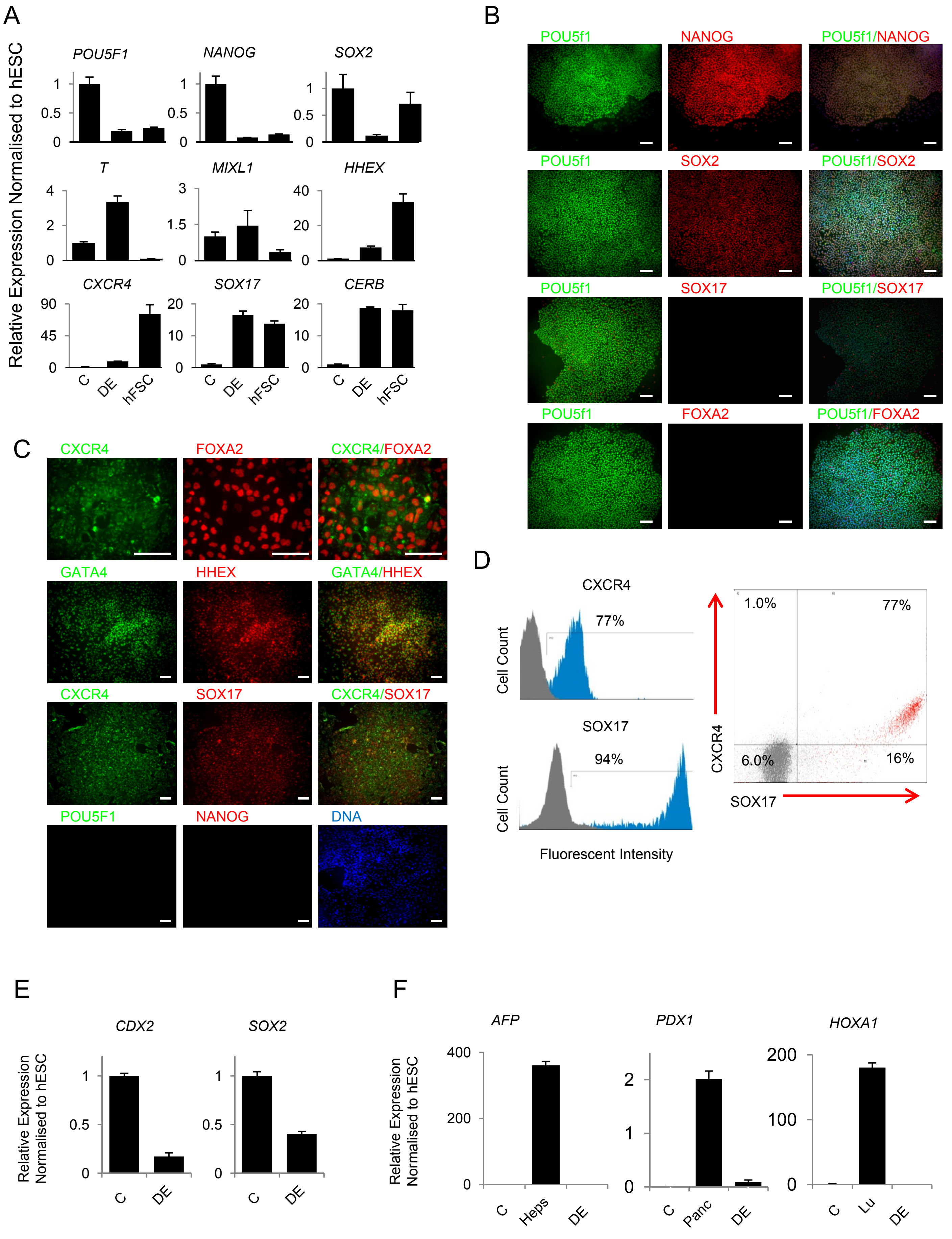

Hannan et al Supplementary Figure 2

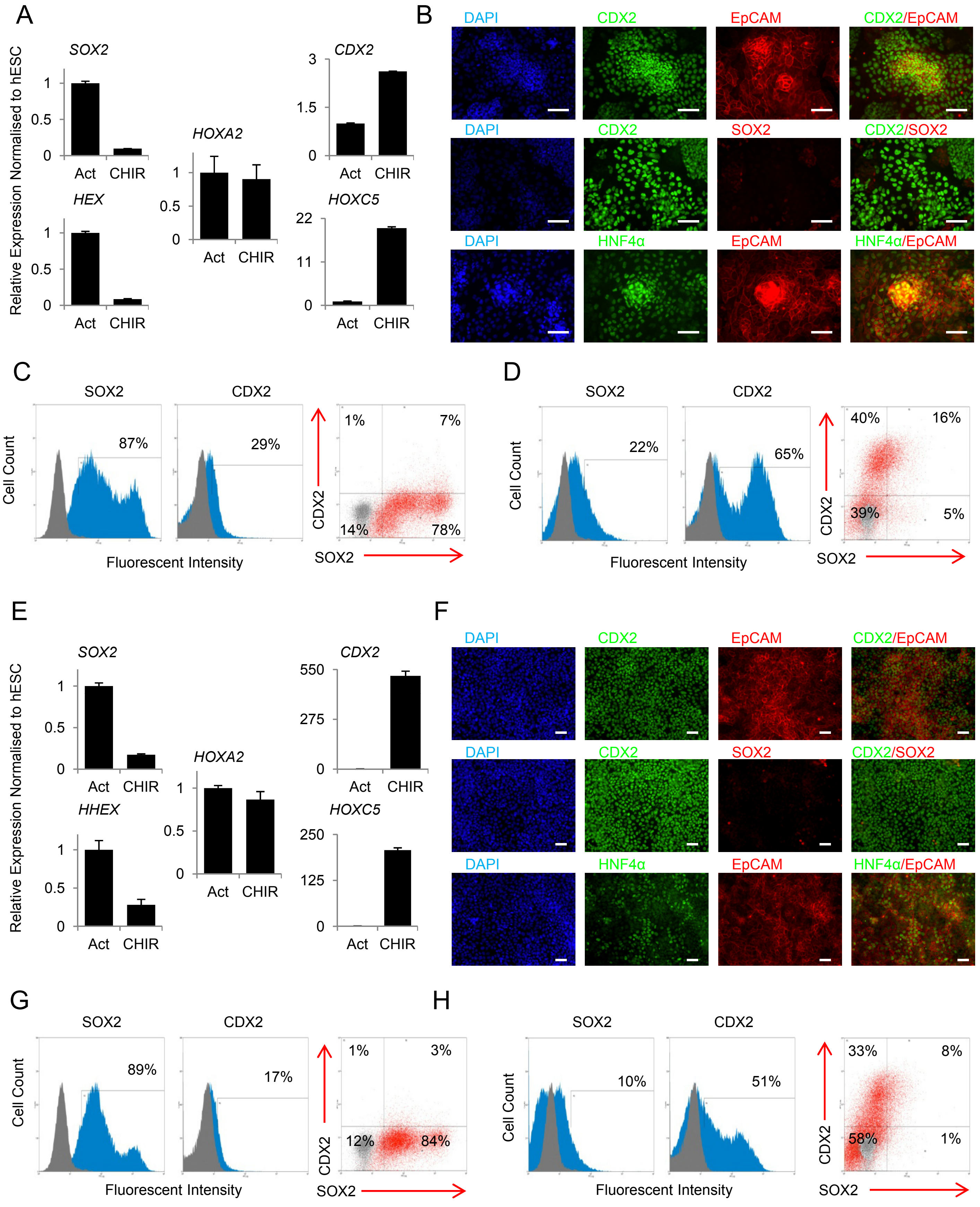

Hannan et al Supplementary Figure 3

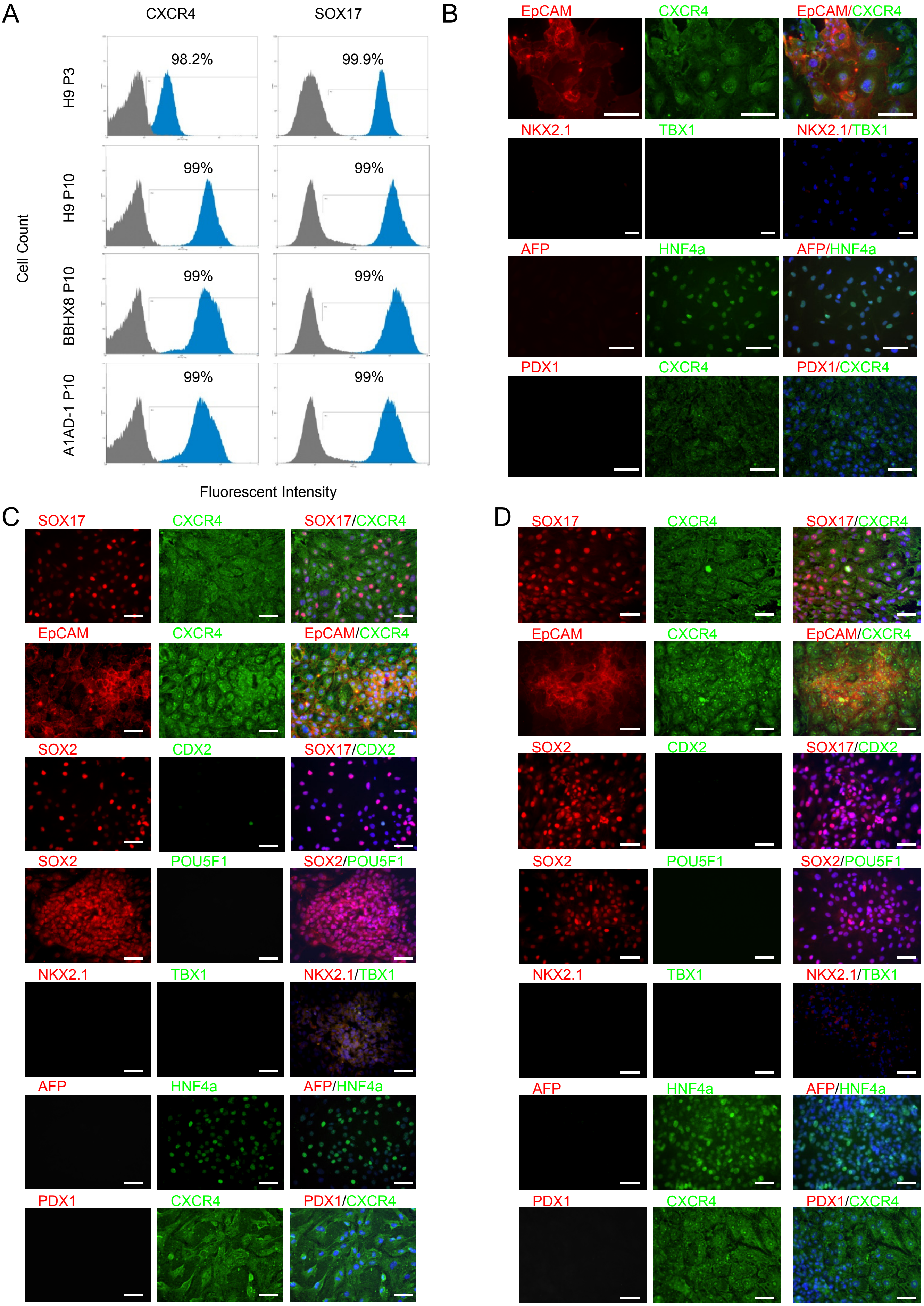

Hannan et al Supplementary Figure 4

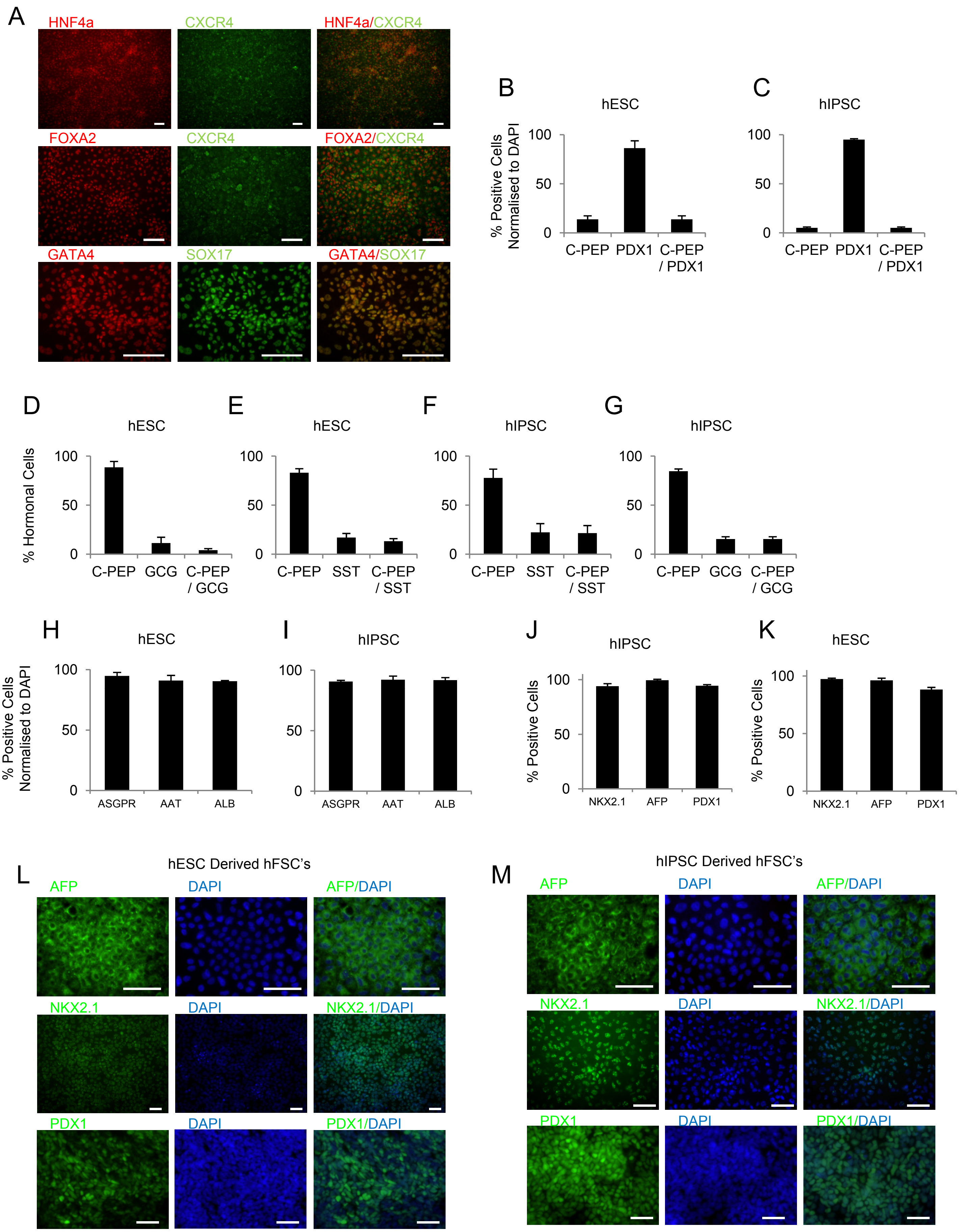

Hannan et al Supplementary Figure 5

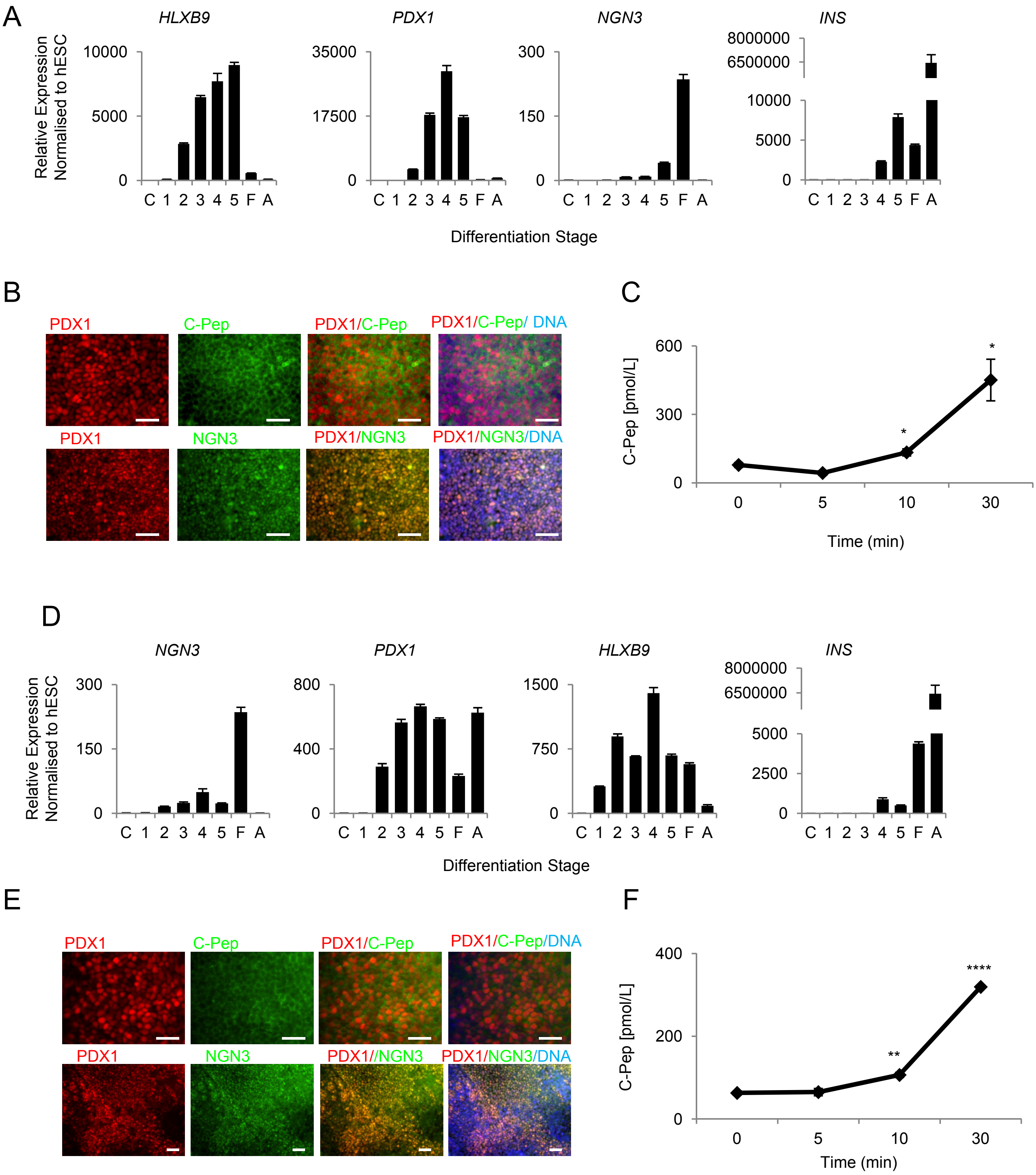

Hannan et al Supplementary Figure 6

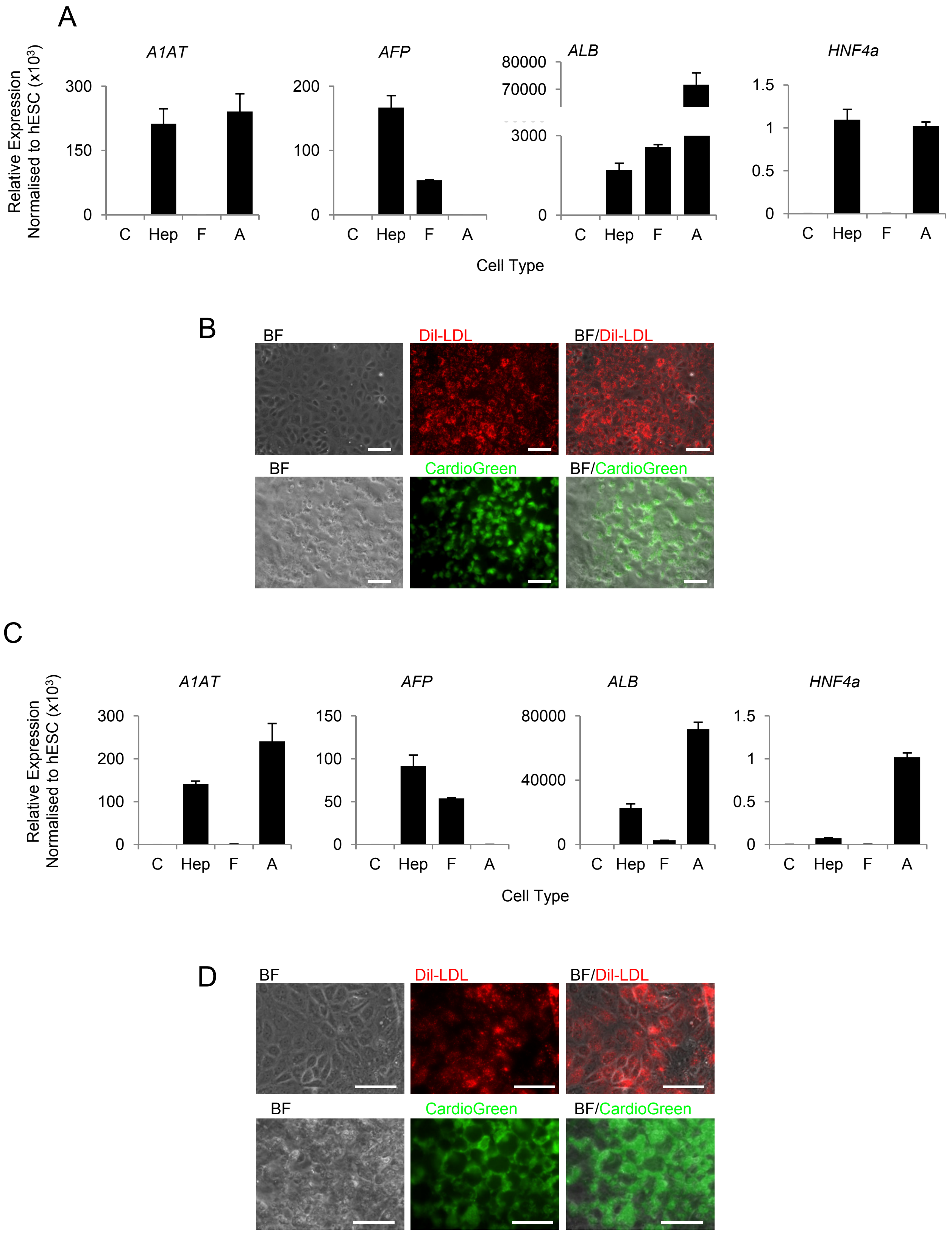

Tissue Potency Assay

hFSC's generated from both hESC and hIPSC's were expanded to P10. At P10 cells were harvested and $1.0x10^5$ cells were mixed in Matrigel and injected under the kidney capsule of NOD-SCID mice. Five mice were used per cell line and cells allowed to grow until significant outgrowth could be felt through abdominal examination (approximately 10 weeks). Mice were sacrificed and outgrowth dissected, outgrowths were then fixed in a 10% formal saline solution for 8 hrs then stored in 70% alcohol.

Cryosectioning and Histology

Outgrowths were immersed in sucrose solution and then mounted in optimal cutting temperature (OCT) compound and stored at -80°C until sectioning. Sections were cut to a thickness of 10µm using a cyrostatmicrotome, mounted on microscopy slides and then stained with haematoxylin and eosin before histological analysis.

RNA Isolation, RT and Q-PCR

RNA was isolated using the GenElute (Sigma-Aldrich) mammalian total RNA isolation kit. Adherent or cell pellets were washed 1x with PBS and then lysed in 350uL of total-RNA lysis buffer. RNA was then purified as per the manufacturer's instructions. DNA digestion was performed using RNAase-free DNase (Sigma) as per the manufacturers recommendations. 500ng of total RNA was reverse transcribed using 500ng total RNA, 0.5uL random primers (Promega) and 1uL of dNTP's (Promega) per reaction. Samples were heated to 65°C for 5 minutes and then placed on ice for a further 5 minutes. 4uL First strand buffer (Invitrogen) + 2uL DTT (Invitrogen) + 1uL RNAse OUT (Invitrogen) + 0.5uL SuperScript II (Invitrogen) was added to each sample and then incubated at RT for 10 minutes, followed by 42°C for 50 minutes and 70°C for 15 minutes.

cDNA from RT-PCR was diluted into a total volume of 500uL of RNAse free water. 5uL of cDNA per reaction was combined with 7.5uL Cyber-Green Sensi mix (Bioline), 0.6uL each of forward primer and reverse primers, and 1.3uL of RNAse free water. PCR was performed using a Stratagene Thermocycler with using 1 cycle at 95°C for 10 minutes, then 40 cycles of 95°C for 30 seconds, 60°C for 30 seconds and 72°C for 30 seconds followed by 1 cycles at 95°C for 1 minutes. A dissociation curve in the range of 55°C - 95°C was produced at the conclusion of each Q-PCR run to confirm the presence of single amplification products. All Q-PCR data show the average of three experiments and error bars show standard error of the mean. hESCs (H9) were used a negative control in all the experiments and error bars represent standard error of the mean (SEM). The internal reference gene used for all QPCR was hydroxymethylbilane synthase (HMBS). For a complete list of primer sequences please refer to supplementary table 2.

DNA Labeling and Hybridisation

Genomic DNA was labeled using the BioPrime DNA Labeling Kit (Invitrogen, 18094-011). 450 ng of each experimental sample and reference were mixed with 60 µl of 2.5X random primer solution (Invitrogen) and nuclease-free water (Ambion, AM9937) to a final volume of 130.5 µl. Samples were denatured at 100 ^{0}C for 10 min before being immediately cooled on ice. After addition 15 µl of 10X dNTP mix (2 mM dATP, 2 mM dGTP, 2 mM dTTP, 1 mM dCTP), 1.5 ul of 1mM Cy5-dCTP (experimental sample) or Cy3-dCTP (reference sample) (GE Healthcare, PA55321) and 3.0 ul of Klenow Fragment (40 U/µl) (Invitrogen), samples were then incubated at 37 ^{0}C for 16 h (overnight). Samples were purified using the PureLink PCR Purification Kit following the manufacturer's protocol (Invitrogen, K3100-01). Labeling efficiency was assessed using the Nanodrop-8000 Spectrophotometer (Thermo Scientific). Samples were hybridised to Agilent 244k human genome arrays following the manufacturer's protocol (G4411B).

Microarray scanning and data analysis

Microarrays were scanned using the Agilent DNA High Resolution Microarray Scanner (Agilent, G2505C) following the manufacturer's protocol. Raw image data was processed using Agilent's Feature Extraction software (v10.7.3.1) and copy number variations were analysed with the Bioconductor packages limma, DNAcopy and CNTools (Smyth, 2004). Graphs were produced using "R".
